# Sweet taste hedonic response in anorexia nervosa: connections with nutritional status and psychopathology

**DOI:** 10.1007/s40519-026-01854-4

**Published:** 2026-04-30

**Authors:** Valentina Pellegatta, Claudia Piciocchi, Francesco Frigerio, Alessandra Arcuri, Antonella Coletta, Laura Tanase, Massimo Pasquini, Armando Cotugno, Caterina Lombardo, Eleonora Poggiogalle

**Affiliations:** 1 Department of Experimental Medicine, Sapienza University, Rome, Italy; 2 Department for the Promotion of Human Sciences and Quality of Life, San Raffaele Open University, Rome, Italy; 3 Department of Psychology, Sapienza University, Rome, Italy; 4 Eating Disorders Unit, “Santa Maria Della Pietà” - ASL Roma 1, Rome, Italy; 5 Department of Human Neurosciences, Sapienza University, Rome, Italy

**Keywords:** Anorexia nervosa, Atypical anorexia, Eating disorders, Sweet taste, Hedonic response, Malnutrition

## Abstract

**Purpose:**

This study aimed at investigating whether taste sensitivity is reduced in patients with anorexia nervosa (AN) and at examining the relationship between sweet taste perception, anthropometric and psychopathological parameters.

**Methods:**

In this case–control study, 15 female patients aged 12–24 years with eating disorders—comprising 13 with AN and 2 with atypical anorexia nervosa (aAN)—and 9 age-matched healthy controls underwent the assessment of taste sensitivity using 5 mL of a 17% sucrose solution on a visual analog scale (VAS) ranging from 0 to 100. Anthropometric parameters, bioimpedance measures and questionnaires were collected.

**Results:**

Patients with AN exhibited significantly lower VAS scores than controls (*p* < 0.05). VAS scores were inversely correlated with BSCL test scores for both the anxiety and depression subscales and the total score (anxiety r_s_ = − 0.489, *p* < 0.05; depression r_s_ = − 0.658, *p* < 0.01; GSI r_s_ = − 0.590, *p* < 0.01), and with the SCL-90 for the same domains (anxiety r_s_ = − 0.529, *p* < 0.05; depression r_s_ = − 0.602, *p* < 0.01; GSI r_s_ = − 0.614, *p* < 0.01). Negative correlations were identified for the EDE-Q subscales of restraint (r_s_ = − 0.429, *p* < 0.05), weight concern (r_s_ = − 0.473, *p* < 0.05), and body shape concern (r_s_ = − 0.501, *p* < 0.05), as well as starvation symptoms on the SSI (r_s_ = − 0.435, *p* = 0.030).

**Conclusion:**

Our findings suggest a blunted hedonic response to sweet taste in patients with AN, particularly among those with poorer nutritional status and higher anxiety and depression traits. Longitudinal studies with larger sample sizes are necessary to evaluate prognostic relevance of assessments of taste sensitivity.

**Level of evidence:**

Level III (evidence obtained from well-designed cohort or case–control analytic studies).

## Introduction

Eating disorders (EDs) encompass a heterogeneous group of psychiatric diseases characterized by persistent alterations in eating behaviors, mechanisms regulating hunger and satiety, and body image perception, resulting in significant health consequences for affected individuals and challenges in weight management.

The gustatory sensory system plays a crucial role in regulating eating behaviors. Gustatory sensitivity encompasses the detection threshold, defined as the ability to distinguish a specific taste stimulus from a neutral one; the recognition threshold, which enables the identification of a specific taste quality and the perception of taste intensity and its hedonic value. Alterations in one or more of these aspects can lead to changes in subjective preferences, food consumption, and appetite regulation [1, 2].

Recent studies have indicated that gustatory sensitivity is compromised in EDs, especially in AN, despite variability in methodologies and sample sizes [2].

A systematic review of 26 studies [2] revealed a significant reduction in gustatory sensitivity in both AN and bulimia nervosa (BN), with hypogeusia prevalence rates of 87% in AN and 84.6% in BN. Following appropriate nutritional intervention and weight restoration, chemosensory function appears to improve; however, in some individuals, the deficit may be irreversible, suggesting individual predisposition. A previous systematic review on gustatory sensitivity in AN similarly demonstrated that patients with AN exhibit reduced gustatory sensitivity, with only partial recovery following weight normalization, while highlighting methodological limitations and inconsistent control of comorbid conditions and potential pharmacological treatments [1].

Using validated instruments such as Sniffin’ Sticks for olfactory assessment and Taste Strips for gustatory evaluation, Dazzi and colleagues [3] identified significant impairments in both sensory systems among individuals with AN and BN. In this case–control study, patients with EDs exhibited lower scores than healthy controls, with diminished gustatory sensitivity, notably for bitterness, and hyposmia, along with an altered olfactory threshold, especially in BN. Regarding hedonic aspects, a few studies on BN pointed out that pleasantness for sweetness was either increased or conserved. Franko and colleagues [4] administered a 40% sucrose solution to patients and found significantly higher gratification scores in patients with BN than in both controls and patients with BN and a history of AN, indicating that clinical history may influence hedonic responses.

According to Moreno and colleagues [5], in AN cognitive and affective processes are intricately linked to sweet tastes and fatty food textures. Patients with AN reported a reduction in perceived self-control and heightened distress after consuming sweets, with a notable increase in thought-shape fusion after tasting. Further exacerbation of responses was observed with fatty and unfamiliar textures, exemplifying the close connection between sensory stimuli and cognitive constructs typical of EDs [5].

A recent systematic review and meta-analysis [6] identified increased exteroceptive capacity in patients with AN and BN. Patients with AN also exhibit markedly heightened taste sensitivity compared to those with BN; conversely, the purging subtypes of AN and BN are characterized by reduced interoception compared to binge eating disorder (BED) and the restricting subtype of AN.

Neuroimaging techniques facilitated the refinement of mechanistic models that relate the gustatory sensory system to reward-learning experiences, interoception, and cognitive control in patients with EDs. Structural and functional imaging consistently identified alterations in the insula, the site of the primary gustatory cortex, and the orbitofrontal cortex and striatal circuits in patients with AN, both during the active phase of the illness and during remission [7, 8]. In women recovering from restricting-type AN, diffusion imaging also documented increased white matter connectivity among the insula, ventral striatum, and orbitofrontal cortex, despite lower microstructural integrity in these areas. This suggests alterations in the taste–reward circuit related to illness duration [9]. Functional multivariate analyses have highlighted lower gustatory classification accuracy in the insula in individuals in the active phase of AN than in patients with AN in remission and healthy controls, consistent with the attenuated neural discriminative capacity for gustatory sensitivity, which could impair adaptive reward coding [10].

Studies based on functional magnetic resonance imaging showed that insular responses to basic sweet stimuli in patients with a controlled nutritional state are diagnosis-specific. In studies where sucrose and sucralose solutions were administered, women in remission from AN exhibited a diminished right anterior insula response to sucrose compared to controls, whereas women recovered from BN showed enhanced insular responses [11]. Ely and colleagues found that, unlike controls whose hunger–satiety mechanisms modulate amygdalar and striatal responses to sweetness, the metabolic state of patients in remission from BN does not influence taste and limbic circuits. Instead, there is more pronounced amygdala activation in the sated state, suggesting a neural correlation with eating beyond satiety during binge episodes [12].

The scientific literature reviewed above consistently suggest that the gustatory sensory system is compromised in EDs, particularly in AN, encompassing perception thresholds, hedonic intensities, and their integration with interoceptive and exteroceptive signals, with variations by disease subtype. Altered reward modulation and satiety are observed in BN. Neuroimaging data describe persistent involvement of the insula, orbitofrontal cortex, striatal circuits, and connectivity changes, indicating both the state and trait components of the illness. These sensory and neurobiological alterations may influence symptom persistence and promote therapeutical interventions focused on hedonic re-education and interoceptive training, thereby allowing treatment personalization by diagnostic subtype. The primary challenge lies in reducing methodological heterogeneity by adopting standardized psychophysical protocols and multimodal approaches [1, 2, 7, 11, 12].

## Methods

In our cross-sectional case–control study conducted from March 2023 to September 2023, a cohort of 24 female participants was recruited and categorized into two groups. Group 1 (cases) comprised 15 individuals diagnosed with an ED (either AN or aAN). The inclusion criteria for this group were female sex, a diagnosis of AN or aAN in accordance with DSM-V criteria, and an age range of 12–24 years. Group 2 (controls) consisted of nine female participants within the same age range, without any diagnosis of ED. Exclusion criteria for both groups included smoking habits, the presence of tongue piercings, and clinical evidence of oral candidiasis. The study protocol received approval from the Ethics Committee, Sapienza University, Rome. Control subjects underwent the same evaluations as the patients in the ED group. All participants, or their legal representatives in the case of minors, provided written informed consent.

Anthropometric measurements were collected based on standardized procedures [13]. Height was measured using a standard stadiometer, to the nearest 0.1 cm, and body weight was assessed using a digital scale, to the nearest 0.1 kg. From these measurements, BMI was calculated, and for participants below 18 years of age, BMI percentile and BMI Standard Deviation Score (BMI SDS) were also determined. MUAC was measured on the non-dominant arm using a flexible tape measure at the midpoint between the lateral margin of the acromion process of the scapula and the inferior margin of the olecranon process of the ulna. A skinfold caliper was employed to assess subcutaneous fat thickness to the nearest 0.1 mm. Each measurement was gathered three times, and the mean value was recorded. The Triceps Skinfold (TSF) and Biceps Skinfold (BSF) were measured at the midpoint of the arm by pinching a vertical skinfold over the triceps and biceps muscles, respectively. The Subscapular Skinfold (SSSF) was assessed approximately two cm below the inferior angle of the scapula. The Suprailiac Skinfold (SISF) was measured above the iliac crest, along the midaxillary line.

Body composition analysis was conducted using a four-electrode bioelectrical impedance analyzer (BIA Akern 101 BIVA PRO and BIA Akern 101 New Edition). Participants underwent bioimpedance assessments while in a fasting state and with an empty bladder. Following a two-minute rest period in the supine position, sensor electrodes were affixed to the right hand at the radio-ulnar joint and to the right foot at the tibio-tarsal joint. Injector electrodes were positioned 5 cm apart near the metacarpophalangeal joint of the third finger and the metatarsophalangeal joint of the third toe, in accordance with the manufacturer's instructions. Resistance (Rz) and reactance (Xc) values were measured, and estimations of Fat-Free Mass (FFM), Fat Mass (FM), Total Body Water (TBW), and Extracellular Water (ECW) were obtained [14, 15].

Additionally, Phase Angle (PA) and Standardized Phase Angle (SPA) were calculated, with the SPA threshold value set at − 1.65°, in alignment with recent studies. A 5 mL dose of a 17% sucrose solution was administered to all participants to evaluate gustatory sensitivity. The solution was prepared by a trained dietitian by dissolving 17 g of sucrose in 100 mL of water at room temperature, storing it in the refrigerator, and returning it to room temperature prior to administration. Each participant was asked to rate the sweetness intensity of the solution using a Visual Analogue Scale (VAS), ranging from 0 to 100. The test was conducted between main meals and following mouth rinsing with water [16].

The self-administered Starvation Symptom Inventory (SSI) consists of fifteen items designed to evaluate the presence of physical and psychosocial symptoms associated with caloric restriction and malnutrition in individuals with EDs over the preceding four weeks. Participants indicated the number of days they experienced each symptom over the past 28 days using a 7-point Likert scale ranging from "never" (0) to "every day" (6) [17]. The total score was obtained as the sum of the scores of the fifteen items, resulting in a possible range of 0–90, with higher scores indicating higher frequency of symptoms.

The Eating Disorder Examination Questionnaire (EDE-Q) is a 28-item self-report instrument designed to assess the type, frequency, and severity of behaviors associated with eating disorders. Items 1–12 and 19–28 utilized a 7-point frequency or intensity scale (0–6). Items 13–18 are employed to identify the presence of binge eating or compensatory behaviors: item 13 investigates binge eating or overeating behaviors; item 14 focuses on binge eating with loss of control; item 15 assesses the frequency of binge eating episodes; item 16 records the number of self-induced vomiting episodes (SIV); item 17 records the use of laxatives; and item 18 assesses the frequency of excessive exercise for compensatory purposes. Three additional items investigated the presence of secondary amenorrhea. The global score (GS) is based on four subscales (restraint, eating concern, shape concern, and weight concern) and quantifies the severity of core ED features [17]. The 53-item self-report Brief Symptom Checklist (BSCL) facilitated the detection of participants’ subjective impairment due to physical and psychological symptoms through nine primary dimensions (somatization, obsessive–compulsive disorder, interpersonal sensitivity, depression, anxiety, hostility/aggression, phobic anxiety, paranoid ideation, and psychoticism) and three global indices. The questionnaire was completed in paper format, with each item scored from 0 (not at all) to 4 (extremely). A total score or scores exceeding 62 in at least two primary dimensions indicated clinically relevant psychological distress [18]. The extended version of the BSCL, the Symptom Checklist-90 (SCL-90), is a self-report questionnaire completed by participants to evaluate a broad range of psychological symptoms. It includes 90 items referring to symptoms experienced in the previous seven days, each rated from 0 (not at all) to 4 (extremely). Based on the responses, ten symptom dimensions were calculated: somatization, obsessive–compulsive symptoms, interpersonal sensitivity, depression, anxiety, phobic anxiety, hostility, paranoid ideation, psychoticism, and sleep disturbance [19]. The score for each dimension was computed as the mean of the answered questions. Mean scores ≥ 1.00 were considered clinically relevant. The Global Severity Index (GSI) was calculated as the average of all answered items and served as a general indicator of psychological distress.

### Statistical analysis

The distribution of the variables was examined for skewness and kurtosis. The Shapiro–Wilk test was used to assess normality. Data are presented as mean ± standard deviation (SD); variables that did not follow a normal distribution were log-transformed. Student’s t-test was used to compare group means. Spearman’s rank correlation coefficient (r_s_) was calculated to assess the correlation between anthropometric variables or psychometric test scores and VAS ratings. The level of statistical significance for all tests was set at *p* < 0.05. Data analyses were performed using SPSS software (IBM SPSS Statistics for Windows, Version 28.0; IBM Corp., Armonk, NY, USA).

## Results

A total of fifteen patients, mean age: 17.07 ± 3.58, were included in the ED group. Out of 15, 13 were diagnosed with AN, while two met the criteria for Atypical Anorexia Nervosa (aAN), but exhibiting reduced body fat associated with secondary amenorrhea. The assisted meal procedure was conducted on 11 of the 15 patients. A total of 13 patients completed the BSCL, and 13 completed the SCL-90; however, only 11 participants successfully completed both assessments. Nine healthy, age- matched female volunteers were included in the control group (mean age: 19.22 ± 2.73). Table 1 displays study participants’ demographic and anthropometric data.

**Table 1:** Sample description

Variables	ED group (n = 15)	Control group (n = 9)	*p*-value
Age (years)*	17.07 ± 3.58	19.22 ± 2.73	0.11
Body weight (kg)	46.11 ± 7.46	60.84 ± 9.66	** < 0.001**
Body weight (kg) (age < 19)	43.91 ± 7.29	58.45 ± 10.44	**0.011**
BMI (kg/m^2^)*	17.08 ± 1.58	23.57 ± 4.43	** < 0.001**
BMI (kg/m^2^) (age < 19)	16.74 ± 0.87	23.13 ± 5.37	0.097
BMI SDS (age < 19)	-1.4 ± 0.7	3.25 ± 1.26	**0.012**
BMI percentile (age < 19)	9.8 ± 6.81	56 ± 30.99	**0.001**
TSF (mm)*	9.02 ± 3.96	17.83 ± 7.23	** < 0.001**
TSF (mm) (age < 19)	7.28 ± 3.58	14.68 ± 3.79	**0.003**
BSF (mm)*	6.15 ± 4.22	12.96 ± 6.65	**0.002**
BSF (mm) (age < 19)	6.49 ± 4.56	11.6 ± 8.48	**0.12**
SSSF (mm)*	8.8 ± 3.36	16.62 ± 8.87	**0.015**
SSSF (mm) (age < 19)	8.41 ± 3.53	13.9 ± 8.53	0.091
SISF (mm)*	10.24 ± 5.8	14.26 ± 7.73	0.082
SISF (mm) (age < 19)	7.88 ± 4.63	13.18 ± 11.21	0.25
MUAC (cm)	21.2 ± 2.5	28.22 ± 3.93	** < 0.001**
Phase angle (°)	5.23 ± 0.54	5.21 ± 0.86	0.94
SPA	-0.43 ± 0.57	-0.03 ± 1.36	0.33
FM (%)	11.4 ± 6.05	30.22 ± 9.14	** < 0.001**
FM (kg)	6.8 ± 3.6	19.3 ± 7.9	**0.001**
FFM (%)	85.9 ± 6	69.8 ± 9.1	** < 0.01**
FFM (kg)	39.33 ± 5.13	41.11 ± 7.34	0.49

ED Group: patients < 19 y.o. (n = 9); patients ≥ 19 y.o. (n = 6). Control Group: patients < 19 y.o. (n = 4); patients ≥ 19 y.o. (n = 5)

*BMI* Body Mass Index, *BMI SDS* Body Mass Index Standard Deviation Score, *BSF* Biceps Skinfold, *ECW* Extracellular Water, *ED* Eating Disorders, *FFM* Fat-Free Mass, *FM* Fat Mass, *MUAC* Mid Upper Arm Circumference, *SISF* Suprailiac Skinfold, *SPA* Standardized Phase Angle, *SSSF* Subscapular Skinfold, *TBW* Total Body Water, *TSF* Triceps Skinfold

*Variables were log-transformed

The ED group was, on average, younger than the control group (17.07 ± 3.58 vs. 19.22 ± 2.73 years); however, this age difference was not statistically significant (*p* = 0.11). In contrast, body weight was significantly lower in the ED group than in the control group for participants over 19 years (46.11 ± 7.46 vs. 60.84 ± 9.66 kg, *p* < 0.001) and those under 19 years (43.91 ± 7.29 vs. 58.45 ± 10.44 kg, *p* = 0.011). The BMI was also significantly lower in the ED group (17.08 ± 1.58 vs. 23.57 ± 4.43 kg/m^2^, *p* < 0.001). In the subgroup over 19 years, BMI remained lower in the ED group (16.74 ± 0.87 vs. 23.13 ± 5.37 kg/m^2^), although this difference was not statistically significant (*p* = 0.097). Among participants aged < 19 years, the ED group exhibited a significantly lower BMI z-score (− 1.4 ± 0.7 vs. 3.25 ± 1.26, *p* = 0.012) and BMI percentile (9.8 ± 6.81 vs. 56 ± 30.99, *p* = 0.001). Skinfold thicknesses were generally reduced in the ED group, with significant differences observed between groups for Triceps Skinfold Thickness (TSF) (9.02 ± 3.96 vs. 17.83 ± 7.23 mm, *p* < 0.001) and age-adjusted TSF (7.28 ± 3.58 vs. 14.68 ± 3.79 mm, *p* = 0.003), for BSF in one entry (6.15 ± 4.22 vs. 12.96 ± 6.65 mm, *p* = 0.002), and for SSSF in one entry (8.8 ± 3.36 vs. 16.62 ± 8.87 mm, *p* = 0.015). However, the BSF in the second entry (6.49 ± 4.56 vs. 11.6 ± 8.48 mm, *p* = 0.12) and SSSF in the second entry (8.41 ± 3.53 vs. 13.9 ± 8.53 mm, *p* = 0.091) did not show significant differences. MUAC was significantly lower in the ED group than in the control group (21.2 ± 2.5 vs. 28.22 ± 3.93 cm, *p* < 0.001). Regarding bioimpedance indices, the PA did not differ between the groups (5.23 ± 0.54 vs. 5.21 ± 0.86 degrees, *p* = 0.94), nor did the standardized phase angle (− 0.43 ± 0.57 vs. − 0.03 ± 1.36, *p* = 0.33). FM was significantly lower in the ED group, both as a percentage (11.4 ± 6.05 vs. 30.22 ± 9.14%, *p* < 0.001) and in kilograms (6.8 ± 3.6 vs. 19.3 ± 7.9 kg, *p* = 0.001). The FFM percentage was significantly higher in the ED group (85.9 ± 6 vs. 69.8 ± 9.1%, *p* < 0.01), while FFM in kilograms did not differ significantly (39.33 ± 5.13 vs. 41.11 ± 7.34 kg, *p* = 0.49).

The psychopathology of EDs, as assessed by the EDE-Q, was significantly elevated in the ED group compared to the control group for the global score (4.75 ± 0.65 vs. 2.12 ± 1.9, *p* = 0.011) and across all subscales. This includes restraint (4.23 ± 1.45 vs 1.84 ± 1.84, *p* = 0.005), eating concern (3.91 ± 1.02 vs 1.56 ± 1.93, *p* = 0.048), weight concern (5.16 ± 0.70 vs 2.47 ± 2.09, *p* = 0.025), and shape concern (5.73 ± 0.32 vs 2.62 ± 2.03, *p* = 0.007). The general symptom burden, evaluated by the BSCL, was also significantly higher in the ED group for the global score (124.36 ± 41.15 vs. 61 ± 39.8, *p* = 0.003) and in clinically relevant domains, with increased levels of depression (2.75 ± 1.17 vs. 1.22 ± 0.93, *p* = 0.005), anxiety (2.66 ± 0.88 vs. 1.41 ± 1.17, *p* = 0.014), and phobic anxiety (1.93 ± 0.7 vs. 0.76 ± 0.47, *p* < 0.001). On the SCL-90, the ED group demonstrated higher scores for depression (2.14 ± 1.11 vs. 1.14 ± 0.87, *p* = 0.041) and anxiety (1.96 ± 1 vs. 1.02 ± 0.97, *p* = 0.048), while the global severity index was higher but not statistically significant (1.76 ± 1 vs. 0.97 ± 0.65, *p* = 0.057), and phobic anxiety showed no significant difference (1.03 ± 1.04 vs. 0.79 ± 0.53, *p* = 0.067). (Table 2).

**Table 2:** Psychometric characteristics of the sample

Variables	ED group (n = 15)	Control group (n = 9)	*p*-value
EDE-Q GS*	4.75 ± 0.65	2.12 ± 1.9	**0.011**
EDE-Q restraint*	4.23 ± 1.45	1.84 ± 1.84	**0.005**
EDE-Q food concern*	3.91 ± 1.02	1.56 ± 1.93	**0.048**
EDE-Q weight concern*	5.16 ± 0.70	2.47 ± 2.09	**0.025**
EDE-Q shape concern*	5.73 ± 0.32	2.62 ± 2.03	**0.007**
BSCL GS	124.36 ± 41.15	61 ± 39.8	**0.003**
BSCL DEP	2.75 ± 1.17	1.22 ± 0.93	**0.005**
BSCL ANX	2.66 ± 0.88	1.41 ± 1.17	**0.014**
BSCL PHOB	1.93 ± 0.7	0.76 ± 0.47	** < 0.001**
SCL-90 GSI	1.76 ± 1	0.97 ± 0.65	0.057
SCL-90 DEP	2.14 ± 1.11	1.14 ± 0.87	**0.041**
SCL-90 ANX	1.96 ± 1	1.02 ± 0.97	**0.048**
SCL-90 PHOB*	1.03 ± 1.04	0.79 ± 0.53	0.067
SSI	51.38 ± 17.16	20.51 ± 17.1	**0.001**

*ANX* Anxiety, *BSCL* Brief Symptom Check-List, *DEP* Depression, *EDE-Q* Eating Disorder Examination Questionnaire, *GS* Global Score, *GSI* Global Score Index, *PHOB* Phobia, *SCL-90* Symptom Check-list, *SSI* Starvation Symptom Inventory

*BSCL and SCL-90 refer to 11 patients in ED group. Variables were log-transformed

**Fig. 1:**
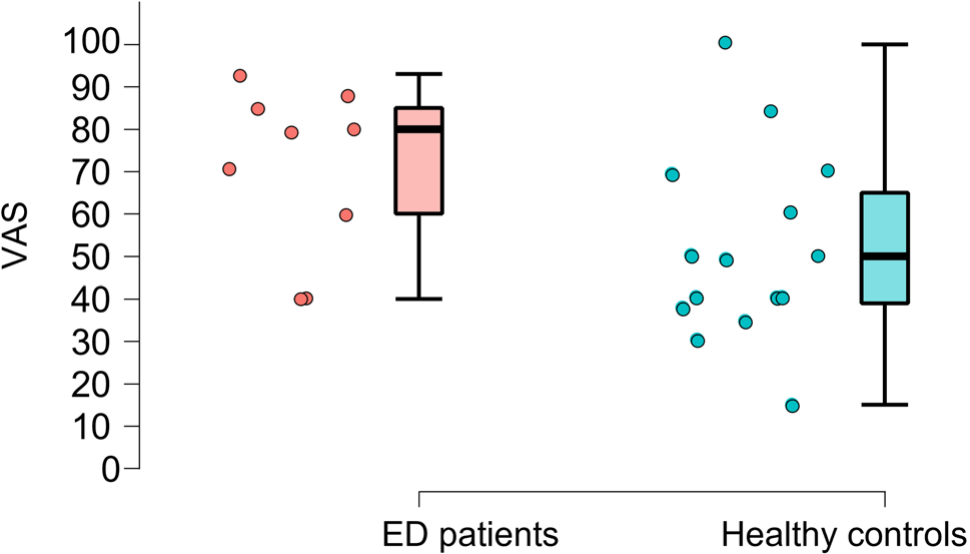
Difference between the ED group and the control group in VAS scores. *VAS* Visual Analogue Scale of Taste Sensivity

VAS scores related to taste perception revealed statistically significant differences between the ED group and the group of healthy participants. Among patients with EDs, hedonic responses to the administration of the 17% sucrose solution showed marked variability. Some patients perceived the taste as excessively sweet and displayed markedly disgusted facial expressions, while others reacted with pleasure and enjoyment. However, 67% exhibited indifference and apathy, with VAS scores equal to or below 50 out of 100, as shown in Fig. 1. Some patients even reported considerable difficulty in assigning a score to the perceived sweetness.

In contrast, among the healthy controls, 22% reported a VAS score ≤ 50%, while 74% gave a score above 50 on the VAS scale.

**Fig. 2:**
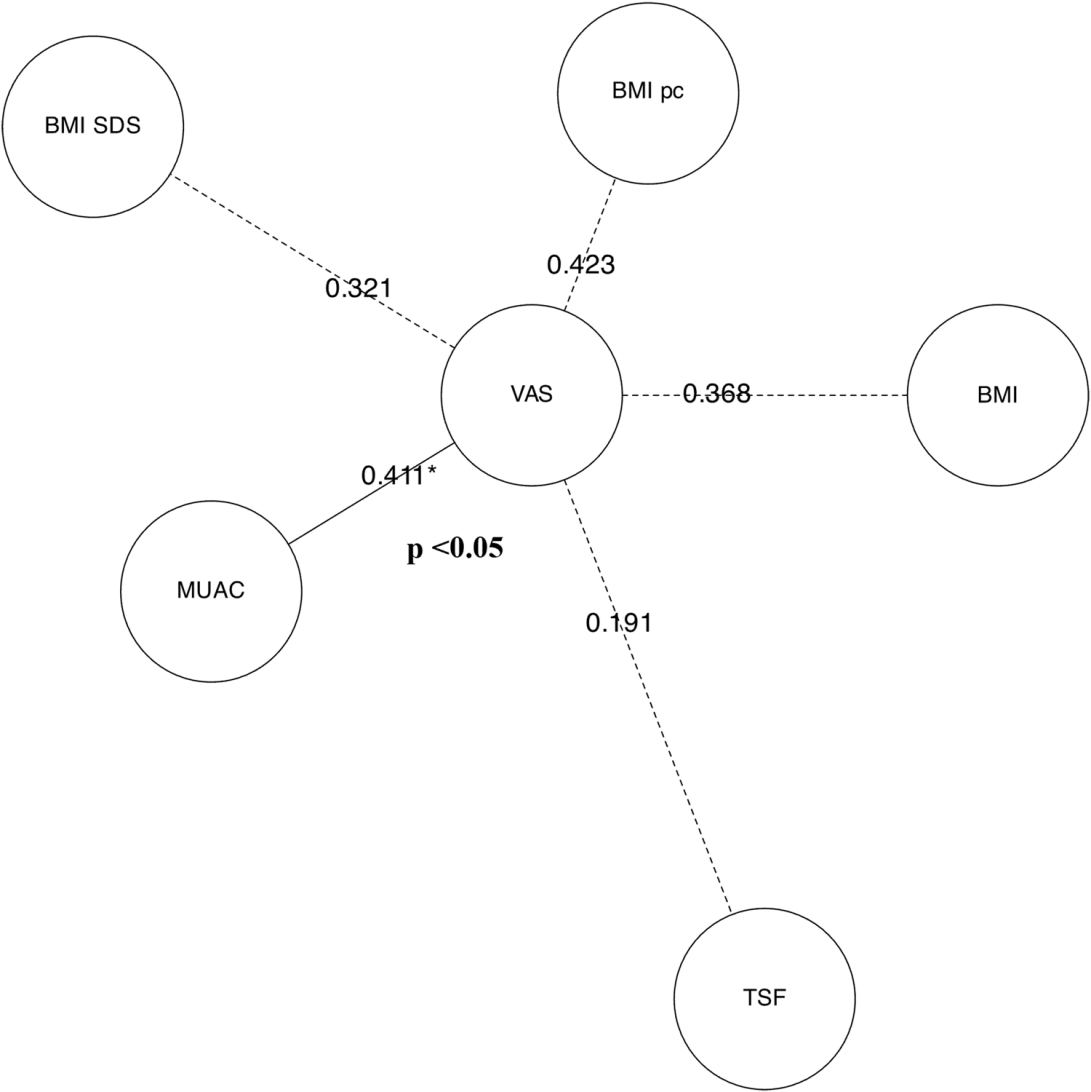
Correlations between VAS and selected anthropometric parameters. *BMI* Body Mass Index (ρ = 0.368), *BMI pc* BMI percentile (ρ = 0.423), *BMI SDS* Body Mass Index Standard Deviation Score or BMI Z-score (ρ = 0.321), *MUAC* Mid Upper Arm Circumference (ρ = 0.411, *p* < 0.05), *TSF* Triceps Skinfold (ρ = 0.191), *VAS* Visual Analogue Scale of Taste Sensitivity

**Fig. 3:**
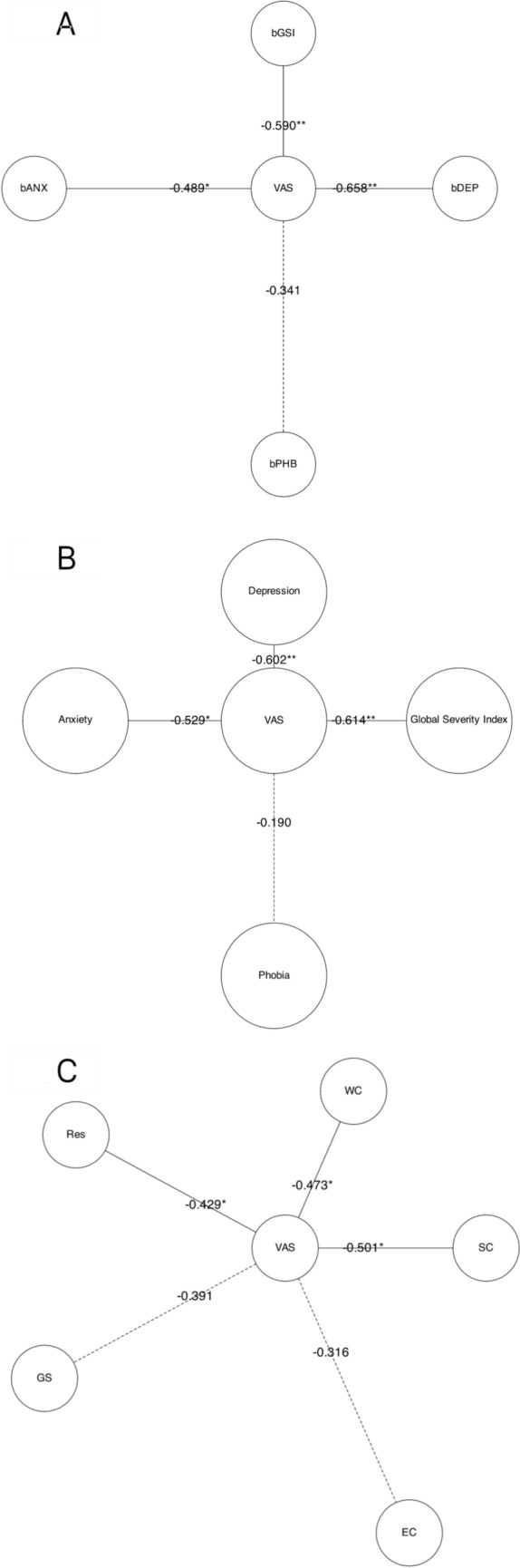
Correlations between VAS and Psychometric tests, 3A. VAS and BSCL, 3B. VAS and SCL-90, 3C. VAS and EDE-Q. *bANX* BSCL Anxiety (ρ = 0.489, *p < 0.05), *bDEP* BSCL Depression (ρ = 0.658, **p < 0.01), *bGSI* BSCL Global Severity Index (ρ = − 0.590, **p < 0.01), *bPHB* BSCL Phobia (ρ = − 0.341), *BSCL* Brief Symptom Check-List, *VAS* Visual Analogue Scale of Taste Sensitivity, *ANX* SCL-90 Anxiety (ρ = − 0.529, *p < 0.05), *DEP* SCL-90 Depression (ρ = − 0.602, **p < 0.01), *GSI* SCL-90 Global Severity Index (ρ = − 0.614, **p < 0.01), *PHB* SCL-90 Phobia (ρ = − 0.190), *SCL-90* Symptom Check List, *VAS* Visual Analogue Scale of Taste Sensitivity, *EC* EDE-Q Eating Concern (ρ = − 0.316), *EDE-Q* Eating Disorder Examination Questionnaire, *GS* EDE-Q Global Scoring (ρ = − 0.391), *Res* EDE-Q Restraint (ρ = − 0.429, *p < 0.05), *SC* EDE-Q Shape Concern (ρ = − 0.501, *p < 0.05), *VAS* Visual Analogue Scale of Taste Sensitivity, *WC* EDE-Q Weight Concern (ρ = − 0.473, *p < 0.05)

**Fig. 4:**
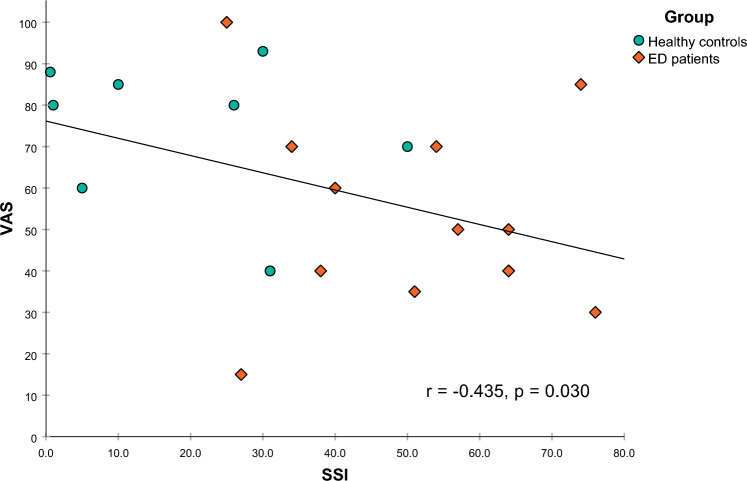
Correlations between VAS and SSI. *SSI* Starvation Symptom Inventory, *VAS* Visual Analogue Scale of Taste Sensitivity

Figures 2, 3 and 4 offer an in-depth analysis of the relationships between anthropometric measurements, psychological evaluations, and VAS scores for taste sensitivity in the study participants.

Figure 2 highlights the positive correlations between VAS scores and anthropometric measurements, with the strongest relationship found in the MUAC, where a larger MUAC correlates with a heightened sweet taste perception (r_s_ = 0.411, *p* < 0.05). Both BMI percentile (r_s_ = 0.423) and BMI SDS (r_s_ = 0.321) also showed positive correlations, whereas BMI (r_s_ = 0.368) displayed a positive trend that was not statistically significant. The correlation with TSF (r_s_ = 0.191) was relatively weak in this study. These results imply that anthropometric factors, especially peripheral muscle mass, as indicated by MUAC, might help preserve taste sensitivity.

In contrast, Fig. 3A displays that VAS scores were negatively correlated with the BSCL subscales. Significant negative correlations were found with anxiety (r_s_ = – 0.489, *p* < 0.05), depression (r_s_ = – 0.658, *p* < 0.01), and the GSI (r_s_ = – 0.590, *p* < 0.01), while the correlation with phobia (r_s_ = – 0.341) was not significant. This negative relationship with the BSCL subscales suggests that a higher psychopathological burden is linked to reduced sweetness perception.

Figure 3B, which explores the correlations between the SCL-90 subscales and VAS scores, confirms a similar pattern: depression (r_s_ = – 0.602, *p* < 0.01), anxiety (r_s_ = – 0.529, *p* < 0.05), and GSI (r_s_ = – 0.614, *p* < 0.01) were all significantly negatively correlated with VAS scores, while phobia (r_s_ = – 0.190) was not significant.

As shown in Fig. 3C, the EDE-Q subscales emphasize the cognitive aspects of EDs. Dietary restraint was negatively correlated with VAS scores (r_s_ = − 0.429, *p* < 0.05), as were weight concerns (r_s_ = – 0.473, *p* < 0.05) and shape concerns (r_s_ = – 0.501, *p* < 0.05). However, the correlations with the global score (r_s_ = − 0.391) and the “eating concern” subscale (r_s_ = − 0.316) were not significant. These findings indicate that concerns about body shape and restrictive behaviors directly affect the ability to perceive sweetness as pleasurable.

Finally, Fig. 4 shows the correlation with malnutrition symptoms (Starvation Symptom Inventory, SSI). This association was negative (r_s_ = – 0.435, *p* = 0.030), suggesting that malnutrition decreases hedonic taste perception. Group analysis further revealed that this pattern was more pronounced in patients than in healthy controls, reinforcing the hypothesis of a specific vulnerability related to eating disorders. Overall, these results paint a coherent picture: on one hand, favorable anthropometric parameters, such as MUAC, enhance gustatory perception; on the other hand, psychopathology (anxiety, depression, global distress), cognitive dimensions assessed by the EDE-Q, and malnutrition symptoms negatively affect taste sensitivity. This dynamic supports the hypothesis that sweet taste is not merely a peripheral epiphenomenon but rather a clinically integrated marker of disorder severity, reflecting both the nutritional and psychological status of the patients.

## Discussion

Our findings suggest that patients with AN exhibit altered processing of sweet taste. These individuals displayed a higher detection threshold for sucralose and differed from controls in their VAS ratings of sweet taste perception, indicating reduced sensory sensitivity and/or altered perceptual appraisal. This pattern may reflect changes in peripheral taste function and/or central gustatory-reward processing, potentially contributing to the persistence of restrictive eating behaviors. It is essential to interpret these results with caution due to the small sample size and the exploratory nature of the analyses.

This diminished perception does not appear to be attributable to an elevated detection threshold but rather to a diminished hedonic salience of sweet stimuli, potentially linked to the partial hypoactivation of the right anterior insula and orbitofrontal cortex [1].This hypothesis is further supported by the observed negative correlation between the severity of anxiety and depressive symptoms and the hedonic response to sweet taste [7, 8, 11].

The findings presented herein are substantiated by the extant literature, notably by several studies encompassed in a recent review article [6]. In a case–control study involving 94 patients with AN [20], identified diminished activation in limbic regions in response to sweet gustatory stimuli, indicating a reduced hedonic component in food-related processing. A subsequent functional magnetic resonance imaging (fMRI) study involving 127 patients with AN and 23 with aAN revealed decreased activity in the orbitofrontal cortex and right anterior insula, regions integral to gustatory processing and reward valuation [21]. Similarly, Frank and colleagues [22] observed reduced activation of the anterior insula in patients with AN in response to sweet stimuli, both subjectively and objectively, compared with healthy controls. Furthermore, a neurobiological model proposed by Kaye [23] posits that dietary restriction in AN activates alternative reward circuits, resulting in a scenario in which food intake is driven not by its hedonic value but by its suppression. Consistent with this, Oberndorfer and colleagues [11] found that in individuals with AN, activation of primary gustatory areas (insula, frontal operculum, and orbitofrontal cortex) is not significantly influenced by hunger or satiety, suggesting a potential disconnection between internal physiological states and affective taste responses.

In our study, we observed persistent alterations in sweet taste perception in some patients with AN, characterized by elevated detection thresholds and low VAS scores. This observation supports the hypothesis that such alterations may represent a stable neurobiological trait rather than a transient state associated with malnutrition, which is reversible upon nutritional rehabilitation and BMI normalization, a notion also supported by Martini and colleagues [20]. Similarly, Monteleone and colleagues [21] reported that taste alterations persist even after weight restoration, suggesting a stable neurobiological basis. Nevertheless, some studies have documented partial improvement in taste sensitivity after refeeding, although normative levels are rarely achieved [24]. Additional evidence suggests that gustatory alterations in AN may reflect a broader profile of atypical sensory processing rather than being limited to objectively measurable psychophysical deficits. Studies utilizing standardized tools, such as the Adolescent/Adult Sensory Profile (AASP) and the Sensory Responsiveness Questionnaire (SRQ), have documented sensory hyperreactivity in individuals with EDs. Brand-Gothelf and colleagues [25], using the SRQ, found increased olfactory and gustatory sensitivity in 20 patients with AN compared to 67 healthy controls and 20 patients with BN. These findings are consistent with a sensory hyperreactivity profile that may contribute to selective avoidance of food. Moreover, the authors suggested that the abnormalities in gustatory and tactile processing identified via the SRQ may represent a cross-cutting phenotype across EDs and serve as a potential target for individualized therapeutic interventions [25].

Additional evidence is provided by the studies conducted by Saure and Zucker, who utilized the AASP and identified significantly elevated scores in the domains of gustatory hypersensitivity and sensory aversion among patients with AN [26, 27]. Although these studies employed different assessment tools compared to our research, their findings corroborate the fundamental observation of altered subjective and objective responses to sweet stimuli. In a seminal study included in a recent review [2], Casper [28] demonstrated pronounced sweet hypogeusia in 20 patients with AN using aqueous sucrose solution. The taste detection threshold for sweetness was significantly higher in patients with AN than in controls. Despite the methodological differences from our study, these results affirm reduced gustatory sensitivity in AN. Similarly, Lacey [29] reported a marked reduction in sweet taste perception using a 0.175% sucrose solution, particularly during the acute phase of AN. Fernandez-Aranda [30] found that over half of the AN patients scored below normal thresholds in sweet taste identification using Taste Strips, further underscoring the clinical significance of these sensory alterations. The alignment between our findings and those of previous studies reinforces the hypothesis that diminished sweet taste sensitivity in AN is not merely an artifact of the assessment method but may represent an intrinsic clinical feature of this disorder. In this context, the integration of both objective (Taste Strips) and subjective (VAS) measures in our study may address a methodological gap in the literature. This dual approach offers the advantage of being simple, reproducible, and quantitative, and is capable of capturing interindividual variability even in clinical settings.

### Strength and limits

In our study, we focus on the findings specific to AN, while briefly acknowledging that prior research suggests sensory phenotypes may vary across ED subtypes, including atypical presentations and binge-purge features, and that this diversity could hold clinical significance [6]. In our AN sample, the hedonic response to a standardized sucrose stimulus was significantly diminished compared to healthy controls, as indicated by lower VAS ratings and a higher proportion of patients assigning low-pleasantness scores. Within the patient group, a higher MUAC was associated with higher hedonic ratings, whereas lower hedonic ratings were linked to a greater affective symptom burden and eating disorder psychopathology, including elevated anxiety and depression scores, increased restraint, and heightened weight and shape concerns. These findings highlight the presence of an AN-related profile characterized by a reduced hedonic valuation of sweetness and its association with both nutritional status and the severity of psychopathology. Given the evidence that sensory processing may differ between restrictive and binge-purge phenotypes, future studies with larger samples should explicitly investigate whether hedonic sweet taste responses vary by clinical subtype, including aAN and purging features, and whether such differences have prognostic or treatment implications [6].

## Conclusions

In this case–control study, individuals diagnosed with AN reported a standardized sucrose stimulus as significantly less pleasurable compared to healthy controls. Among those with anorexia nervosa, a reduced enjoyment of sweet tastes was correlated with poorer nutritional health and more severe affective and eating disorder-related psychopathology. Although the clinical utility was not assessed, these findings suggest that brief hedonic evaluations of sweet taste could be a valuable complement to anthropometric and psychometric assessments. They warrant further investigation in larger longitudinal studies, including atypical and binge-purge cases, to ascertain their prognostic and therapeutic significance.

### What is already known on this subject?

Many patients with AN perceive sweet taste as less pleasant, as confirmed by neuroimaging studies. Alterations of the gustatory sensory system may play a crucial role in the persistence of eating disorder symptoms. More research is needed to determine the relationship between taste perception and organic and psychological parameters in AN.

### What this study adds?

In this study, reduced pleasantness was associated with higher levels of anxiety and depression and more pronounced starvation symptoms, whereas a better mid-upper arm circumference, an indirect indicator of nutritional status, correlated with greater pleasantness. Simple assessments of gratification related to sweet taste could thus assist clinicians during nutritional and psychological interventions, as well as in the recovery phase, to monitor their effectiveness.

## Data Availability

The datasets generated during and/or analysed during the current study are available from the corresponding author on reasonable request.
